# Intrinsic Kidney Pathology Following COVID-19 Infection in Children and Adolescents: A Systematic Review

**DOI:** 10.3390/children9010003

**Published:** 2021-12-22

**Authors:** Henry H. L. Wu, Mohan Shenoy, Philip A. Kalra, Rajkumar Chinnadurai

**Affiliations:** 1 Department of Renal Medicine, Lancashire Teaching Hospitals NHS Foundation Trust, Preston PR2 9HT, UK; 2 Faculty of Biology, Medicine and Health, University of Manchester, Manchester M13 9PG, UK; Philip.Kalra@nca.nhs.uk (P.A.K.); Rajkumar.Chinnadurai@nca.nhs.uk (R.C.); 3 Department of Paediatric Nephrology, Royal Manchester Children’s Hospital, Manchester University NHS Foundation Trust, Manchester M13 9WL, UK; Mohan.Shenoy@mft.nhs.uk; 4 Department of Renal Medicine, Salford Royal Hospital, Northern Care Alliance Foundation Trust, Salford M6 8HD, UK

**Keywords:** intrinsic kidney pathology, COVID-19, SARS-CoV-2, children, adolescents

## Abstract

*Introduction:* COVID-19 infections resulting in pathological kidney manifestations have frequently been reported in adults since the onset of the global COVID-19 pandemic in December 2019. Gradually, there have been an increased number of COVID-19-associated intrinsic kidney pathologies in children and adolescents reported as well. The pathophysiological mechanisms between COVID-19 and the onset of kidney pathology are not fully known in children; it remains a challenge to distinguish between intrinsic kidney pathologies that were caused directly by COVID-19 viral invasion, and cases which occurred as a result of multisystem inflammatory syndrome due to the infection. This challenge is made more difficult in children, due to the ethical limitations of performing kidney biopsies to reach a biopsy-proven diagnosis. Although previous systematic reviews have summarized the various pathological kidney manifestations that have occurred in adults following acute COVID-19 infection, such reviews have not yet been published for children and adolescents. We describe the results of a systematic review for intrinsic kidney pathology following COVID-19 infection in children and adolescents. *Methods:* A systematic literature search of published data up until 31 October was completed through the Preferred Reporting Items for Systematic Reviews and Meta Analyses (PRISMA) guidelines. Research articles reporting new-onset or relapsed intrinsic kidney pathology in children or adolescents (≤18 years) following acute COVID-19 infection were included for qualitative review. COVID-19 infection status was defined by a positive result from a RT-PCR, or nuclear antibody testing. Only full-text articles published in the English language were selected for review. *Results:* Twenty-nine cases from fifteen articles were included in the qualitative synthesis of this systematic review. Nephrotic syndrome, as an umbrella condition, appeared as the most frequently observed presentation (20 cases) with disease remission noted in all cases with steroid treatment. Other cases included numerous glomerulonephritides, such as acute necrotizing glomerulonephritis, MPO vasculitis and collapsing glomerulopathy, and thrombotic microangiopathies, such as aHUS. For patients with transplanted kidneys, T-cell-mediated rejection and mild tubular interstitial infiltration were noted following testing positive for COVID-19. There were no mortalities reported in any of the included cases, although two patients remained dialysis dependent at hospital discharge. *Conclusion:* This systematic review highlights the various intrinsic pathological kidney manifestations in children and adolescents as a result of acute COVID-19 infection. The clinical timeline and presentation of these cases support the mechanistic hypothesis between COVID-19 infection and the onset of intrinsic kidney pathologies within this context. The progressive introduction of vaccination programs for children and adolescents may hopefully reduce the severity of COVID-19-associated illnesses, and pathological kidney manifestations in this population.

## 1. Introduction

The coronavirus disease (COVID-19) that began in 2019 has spiraled into a global pandemic, continuing up to this day, since the identification of the first severe acute respiratory syndrome coronavirus 2 (SARS-CoV-2) case in Wuhan, China, in December 2019 [1,2]. Whilst data relating to the epidemiology, pathophysiology, risk factors and prognosis of adults with kidney histopathology following acute COVID-19 infection are becoming more well-established, there remains a sizable knowledge gap in our understanding of intrinsic pathological kidney manifestations in children following acute COVID-19 infection [3]. This is likely explained by the fact that children and adolescents only form about 1–2% of all COVID-19 cases reported globally [4].

A novel syndrome, currently termed multisystem inflammatory syndrome in children (MIS-C), has been recognized by clinicians in the United Kingdom since April 2020. It describes previously healthy children presenting with severe systemic inflammatory syndrome following testing positive for a concurrent or recent COVID-19 infection [5]. MIS-C has been shown to be significantly associated with acute kidney injury (AKI) presentations in children, with an incidence of up to 60% being reported in observational studies [6,7]. Active research is being pursued to differentiate pediatric presentations of AKI from MIS-C following COVID-19 infection, and AKI from direct intrinsic kidney injury as a result of primary COVID-19 infection, with overlapping clinical features between these two entities [8]. There is a growing effort to elicit the specific mechanisms of intrinsic kidney pathophysiology following acute COVID-19 infection. Determining these mechanisms could be challenging, due to the ethical limitations of pursuing routine kidney biopsy for children, and the lack of an accurate non-invasive diagnostic test at present [8,9].

Isolated case reports and case series have been published since the onset of the COVID-19 pandemic, describing newfound or relapsed cases of podocytopathy, glomerular disease and other intrarenal pathologies in children following acute COVID-19 infection. Some of these reported cases are kidney-biopsy-proven, whereas others are empirical diagnoses determined through past medical history relating to kidney disease, non-invasive investigations, and responses to treatment.

As of 31 October 2021, there have been no systematic reviews published to summarize the findings of kidney histopathology following acute COVID-19 infection in children. Here, we provide a systematic clinical review of the current literature to delineate the range of intrinsic kidney pathologies that have manifested following acute COVID-19 infection in children.

## 2. Materials and Methods

### 2.1. Eligibility Criteria

All research articles reporting new-onset or relapsed intrinsic kidney pathology in children or adolescents (≤18 years) following acute COVID-19 infection were included. COVID-19 infection status was defined by a positive result from a real-time reverse transcription polymerase chain reaction (RT-PCR), or nuclear antibody testing. Articles for inclusion described intrinsic kidney pathology in both native and transplanted kidneys. Only full-text articles published in the English language were selected for review. Only studies published before 31 October 2021 were included in this review.

### 2.2. Search Strategy and Study Selection

**Figure 1:**
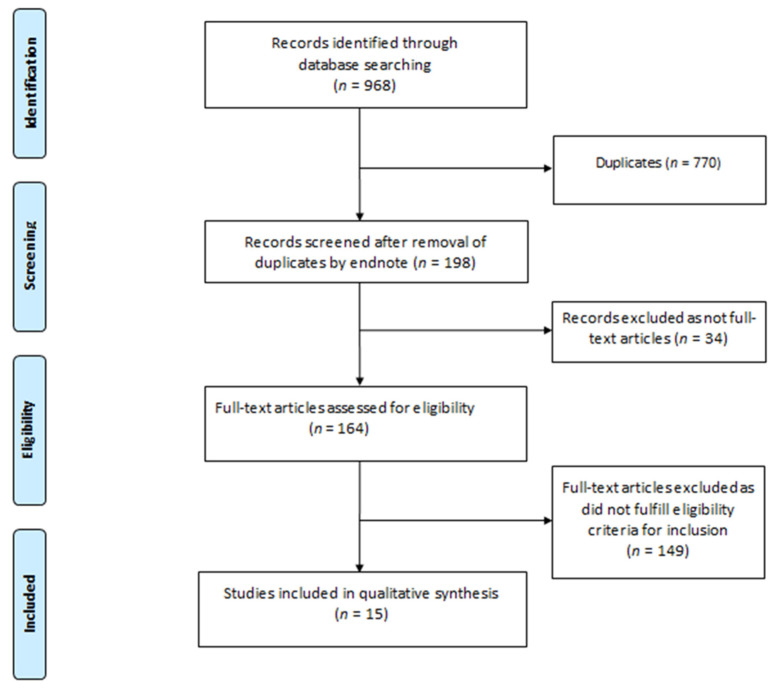
PRISMA Flow Diagram.

A systematic literature search was conducted by two independent authors (H.H.L.W. and R.C.) in the following databases: “PubMed”, “Web of Science”, “EMBASE” and “Medline-ProQuest”. The search terms incorporated the following: “COVID-19” AND “Kidney Histopathology” AND “Children”; “SARS CoV-2” AND “Kidney Histopathology” AND “Children”; “COVID-19” AND “Renal Histopathology” AND “Children”; “SARS-CoV-2” AND “Renal Histopathology” AND “Children”; “COVID-19” AND “Kidney Manifestations” AND “Children”; “COVID-19” AND “Renal Manifestations” AND “Children”; “SARS-CoV-2” AND “Kidney Manifestations” AND “Children”; “SARS-CoV-2” AND “Renal Manifestations” AND “Children”; “COVID-19” AND “Nephrotic Syndrome” AND “Children”; “SARS-CoV-2” AND “Nephrotic Syndrome” AND “Children”; “COVID-19” AND “Glomerulonephritis” AND “Children”; “SARS-CoV-2” AND “Glomerulonephritis” AND “Children”; “COVID-19” AND “Kidney Histopathology” AND “Adolescents”; “SARS CoV-2” AND “Kidney Histopathology” AND “Adolescents”; “COVID-19” AND “Renal Histopathology” AND “Adolescents”; “SARS-CoV-2” AND “Renal Histopathology” AND “Adolescents”; “COVID-19” AND “Kidney Manifestations” AND “Adolescents”; “COVID-19” AND “Renal Manifestations” AND “Adolescents”; “SARS-CoV-2” AND “Kidney Manifestations” AND “Adolescents”; “SARS-CoV-2” AND “Renal Manifestations” AND “Adolescents”; “COVID-19” AND “Nephrotic Syndrome” AND “Adolescents”; “SARS-CoV-2” AND “Nephrotic Syndrome” AND “Adolescents”; “COVID-19” AND “Glomerulonephritis” AND “Adolescents”; “SARS-CoV-2” AND “Glomerulonephritis” AND “Adolescents”. The articles were screened by H.H.L.W. and R.C. for relevance and duplicate publications were removed. Duplicate screening and the eligibility check was performed by both H.H.L.W. and R.C. The study selection process was carried out using the Preferred Reporting Items for Systematic Reviews and Meta Analyses (PRISMA) guidelines (Figure 1). Amongst 164 full-text articles assessed for eligibility for inclusion, 149 articles did not fulfill the inclusion criteria, and were hence excluded from the full-text review. Fifteen articles were included and proceeded to qualitative synthesis.

### 2.3. Data Extraction

If available, data including patient demographics (age, sex and ethnicity), co-morbidities, clinical presentation, kidney parameters at the time of presentation (serum creatinine, serum albumin, presence and degree of proteinuria and hematuria), information on whether kidney biopsy was performed, treatment received following diagnosis and clinical outcome following treatment, were extracted from the included articles. These data are described in the results section of this article, and also presented in tabular form.

### 2.4. Study Registration

A pre-defined review protocol was registered at the PROSPERO international prospective registry of systematic reviews, under registration number CRD42021289662.

## 3. Results

### 3.1. New-Onset and Relapsed Nephrotic Syndrome

A total of 20 cases involving children or adolescents presenting with either new-onset or relapsed nephrotic syndrome following acute COVID-19 infection were included in our systematic review (Table 1) [10,11,12,13,14,15,16,17,18]. In 13 of these 20 cases, there was a relapse of a previously known nephrotic syndrome, and detailed data in relation to the patient demographics, clinical presentation, investigation results and outcome were incomplete in the majority of these reported cases. Such data were also complete amongst the seven patients of which this was the first presentation of nephrotic syndrome. The median age was 6.5 (range 3 to 15) years, and there was a predominance of the male gender (five cases). A diverse distribution of ethnic origin was observed. None of the 20 patients in these nephrotic cases had kidney biopsy during hospital admission, and diagnoses were made empirically, based on clinical presentation of systemic edema (abdominal distension, facial swelling, and/or lower limb edema) and nephrotic-range proteinuria. Standard treatment included oral steroid therapy in addition to supportive treatment (albumin infusion and diuresis). All of the reported cases achieved remission of nephrotic syndrome following acute treatment.

### 3.2. Glomerulonephritis and Hemolytic-Uremic Syndrome

Glomerulonephritis and hemolytic-uremic syndrome in children and adolescents associated with acute COVID-19 infection included in our systematic review are presented in Table 2.

Basiratnia et al. [19] reported two cases of adolescent boys, aged 16 and 17 years, presenting with AKI in association with a concurrent acute COVID-19 infection. Urinalysis displayed frank hematoproteinuria on presentation, and kidney biopsies were performed in both cases. Histopathological evaluation of the kidney biopsies showed acute necrotizing glomerulonephritis with fibrocellular crescents. Anti-neutrophil cytoplasmic antibodies (ANCAs) and anti-glomerular basement membrane (anti-GBM) results were not reported. Both patients required acute hemodialysis and were commenced on high-dose steroid therapy. The 16-year-old boy achieved full recovery of kidney function and remained dialysis independent. However, the 17-year-old boy required maintenance dialysis following discharge.

A 17-year-old boy with recent hospital admission in relation to COVID-19 pneumonia (receiving dexamethasone, remdesivir and azithromycin during that admission) was re-admitted one month following his initial hospitalization [20]. He re-presented with AKI and frank hematoproteinuria, which was treated as pre-renal AKI and the patient achieved complete kidney function recovery. A month after this second acute presentation, he further re-presented with acute shortness of breath and cough symptoms, although his COVID-19 RT-PCR test was negative when rechecked. Computed tomography of the chest was suggestive of pulmonary hemorrhage, after which bronchoalveolar lavage confirmed this finding. Serum testing revealed severe AKI, and further immunology screening detected positivity for the perinuclear anti-neutrophil cytoplasmic antibody (P-ANCA) and myeloperoxidase (MPO) antibodies. With vasculitis being the presumed diagnosis, a kidney biopsy was performed which revealed necrotizing glomerulonephritis. The patient required acute hemodialysis, and was treated with plasmapheresis, steroid therapy and cyclophosphamide. Full resolution of AKI was achieved during this hospital admission, and he remained dialysis independent following discharge.

Relapse of recurrent anti-factor H antibody-associated atypical hemolytic uremic syndrome (aHUS) with an underlying complement factor H-related protein mutation was found in a 10-year-old boy with a positive COVID-19 infection diagnosis through nuclear antibody testing [21]. The patient presented with frank proteinuria and commenced oral steroid therapy and mycophenolate mofetil during his hospital admission. He also received two doses of intravenous rituximab, and required intravenous immunoglobulin. The patient was discharged with end-stage kidney disease, requiring maintenance hemodialysis and regular oral steroid therapy.

### 3.3. Transplant Intrinsic Kidney Pathologies

Regarding children and adolescents with transplanted kidneys (Table 3), a case of collapsing glomerulopathy (CG) with concurrent chronic antibody-mediated rejection, and another case of T-cell-mediated rejection, were reported in association with acute COVID-19 infection for two adolescents aged 15 and 16 years, respectively [22,23]. In both of these cases, the patients required meticulous optimization of their immunosuppression medications, alongside supportive medical management to reduce the risk of further COVID-19-associated complications. Another two cases in patients with recent kidney transplants were found to have COVID-19 RT-PCR positive test results on day 2 and day 105 after their operations, respectively [24]. Both patients did not display clear-cut kidney pathological manifestations following the COVID-19 infection diagnosis, with the kidney biopsy finding < 10% tubular interstitial infiltration in one patient, and microcalcifications in the other. Both patients remained largely asymptomatic following, and were managed supportively.

## 4. Discussion

Numerous intrinsic kidney pathologies were described following acute COVID-19 infection in children and adolescents within this systematic review, from which a total of 28 cases from 15 published articles were included in qualitative analysis. Nephrotic syndrome appeared as the most frequently reported clinical presentation. Nephrotic syndrome is a common kidney pathology observed in children and adolescents, characterized by minimal change disease in the majority (more than 80%) [25,26]. It is defined by the inability to restrict urinary protein loss, due to alterations of perm-selectivity in the capillary walls of the glomerulus, as a result of podocyte injury. Nephrotic-range proteinuria is recognized as the equivalent of 3.5 g or more of protein identified from a 24-h urine sample collection (urine protein-creatinine ratio > 300 mg/mmol), and childhood nephrotic syndrome tends to be selective towards albuminuria [27]. In children and adolescents, approximately 95% of nephrotic syndrome presentations are idiopathic, with the remaining 5% secondary to causes such as viral diseases (e.g., Parvovirus B19, Human Immunodeficiency Virus (HIV), Hepatitis B and C), inflammatory conditions (e.g., Juvenile Idiopathic Arthritis) or rare conditions such as Amyloidosis and Henoch–Schonlein Purpura [28,29,30,31,32,33]. Although COVID-19-associated minimal-change nephrotic syndrome is increasingly reported, its epidemiology in comparison with those minimal change cases induced by other viral infections remains unclear at this point in time. The mechanisms of how COVID-19 might induce nephrotic syndrome have been postulated in several adult studies, but there is very limited data for comparison in children and adolescents. A study conducted in China by Su et al. [34], with post-mortem kidney biopsy samples, found SARS-CoV-2 virion particles in podocytes with effaced foot-processes, suggestive of direct podocytopathic injury. Another report found evidence of tubuloreticular inclusions, often a marker of viral replication and marked interferon production within endothelial cells in the glomerulus [35]. These results have been disputed, with suggestions that the ultrastructural histological findings were actually normal subcellular structures, such as clathrin-coated vesicles and multivesicular bodies [36,37]. An alternative perspective on the mechanism of COVID-19-induced nephrotic syndrome advocates that it is mediated by multiple immunological pathways. Results from basic science studies during the early days of the pandemic suggest tissue damage from SARS-CoV-2 virus is defined by the generation of a cytokine storm [38]. It is believed that podocytopathy can be triggered by the cytokine storm generating an immunological milieu with excessive production of Th2-generated cytokines. Though there are ethical controversies to consider, it was a limitation that there were no kidney biopsy-proven histopathologies from the nephrotic syndrome presentations reviewed. In steroid-sensitive nephrotic syndrome, which represents almost 99% of all idiopathic nephrotic syndrome cases in children aged 1–12 years, kidney biopsy is not usually performed unless a child does not achieve remission following a 4-week course of steroids [39]. Mechanistic associations between COVID-19 infection and nephrotic syndrome in children and adolescents may have been further ascertained with evaluation of kidney biopsy findings.

Various glomerulonephritides have been reported in children and adolescents following COVID-19 infection. CG is a variant of Focal Segmental Glomerulosclerosis (FSGS), characterized by glomerular tuft collapse, segmentally or globally [40,41,42]. CG has previously been reported to associate with multiple infections and inflammatory conditions, namely HIV and systemic lupus erythematous [43]. Previous reviews evaluating COVID-19 induced glomerular disease in adults suggest CG to be amongst the most prevalent histopathological presentations [3,44]. The pathogenesis of CG is currently believed to be a multifactorial process associated with both direct viral invasion of glomerular structures and cytokine release [45,46]. Adult individuals of Afro-Caribbean ethnicity, and those carrying the APOL1 high-risk genotype have been shown to be at higher risk of CG following acute COVID-19 infection, due to stimulation of interferon production encouraging APOL1 gene expression [45,46].

AKI secondary to acute necrotizing glomerulonephritis and vasculitis in acute COVID-19 infection is hypothesized to be caused by glomerular hypoperfusion and tubular necrosis, leading to fibrinoid necrosis within the glomerulus and arterial walls of the intrarenal vessels [47,48]. Neutrophil extracellular trap (NET) formation, as part of the innate inflammatory process of acute COVID-19 infection, has been hypothesized to play a major role in ANCA antibody formation, by affecting an individual’s immunotolerance during the acute inflammatory state of COVID-19 infection [49]. It is proposed that NET formation is the ultimate source of presentation of MPO and proteinase 3 antigen within this context [49].

In addition, aHUS following COVID-19 infection is thought to be caused by a prothrombotic state, as a result of complement-mediated inflammation and thrombotic microangiopathy-associated processes [50,51]. Models of mice with complement 3 deficiency have shown reduced respiratory distress and pulmonary inflammation, after infection with severe acute respiratory syndrome coronavirus (SARS-CoV), whilst Middle Eastern respiratory syndrome coronavirus (MERS-CoV) infection and elevated levels of C5a and C5a-9 complexes have also been previously demonstrated in mice models [52,53]. These limited findings provide evidence of the significant role played by the complement system in generating hyperinflammatory responses during coronavirus-associated infections. Further work needs to be conducted to validate the role of complement in COVID-19, and its correlation with the onset of thrombotic microangiopathies.

Poorer outcomes following COVID-19 infection in children and adolescents with prior kidney transplantation are expected due to chronic immunosuppression and other co-existing co-morbidities, such as diabetes, mellitus and hypertension, now recognized as significant risk factors for mortality following acute COVID-19 infection [54,55,56]. Due to the lack of reported cases, the precise mechanisms of how acute COVID-19 infection causes kidney transplant rejection and other transplant-associated conditions in children and adolescents requires further exploration. The lack of reported cases may be attributed to the fact that during the first wave of the COVID-19 pandemic, many children with transplanted kidneys were shielding with schools being closed. In our region, the North West of the UK, this has resulted in an extremely low incidence of COVID-19 within this population due to infrequent testing of COVID-19 status.

There remain knowledge gaps in our understanding of the mechanisms associating intrinsic kidney pathologies and COVID-19 infection. Incomplete data regarding kidney histopathology due to limitations in performing kidney biopsy and other invasive investigations for many cases involving children and adolescents has contributed to this. There was a published case series of AKI presentations with proteinuria and hematuria in children and adolescents, following positive COVID-19 PCR results [57]. However, these cases were not included in our systematic review, due to the lack of diagnoses provided from these reports. In other instances, it is difficult to clearly delineate and distinguish the differences between intrinsic kidney pathology caused by MIS-C, and cases that result from a direct viral invasion from COVID-19. Questions remain regarding whether genuine cases of COVID-19 infection-related kidney disease have been missed due to mistimed testing. There have been cases where the appearance of new-onset kidney pathology could not be directly attributed to acute COVID-19 infection, although the patient’s clinical presentation and timeline supported the likelihood of COVID-19-associated illness [58]. Whilst the co-morbidity status of children and adolescents may associate with greater risks of developing these manifestations following COVID-19 infection, in particular for relapsed rather than new-onset kidney disease, further work is required to determine the mechanisms by which acute COVID-19 infection induces renal disease, and to what extent these presentations are impacted by the presence of other confounding factors. The variations in clinical and pathological intrinsic kidney manifestations are currently difficult to explain, due to the relative novelty and lack of case numbers.

## 5. Conclusions

Our systematic review has been able to pool the currently reported cases of new-onset and relapsed intrinsic kidney pathology in children and adolescents following COVID-19 infection, and summarises the purported mechanisms behind the links between COVID-19 and the onset of intrinsic kidney disease. Although the adverse renal complications of COVID-19 vaccination are recognized in adults, these are very rare [59]. The progressive introduction of COVID-19 vaccination programs for children and adolescents may ultimately lead to decline of COVID-19-associated severe illness and kidney pathological manifestations within the pediatric population.

## Figures and Tables

**Table 1:** Demographics and outcomes of children and adolescents with new-onset and relapsed nephrotic syndrome following acute COVID-19 infection.

Author(s) and Country of Report	Age (yrs)	Sex	Ethnicity	Comorbidities	New-onset or Relapse	Clinical Presentation	Presentation Creatinine (mg/dL)	Presentation Proteinuria (g/day)	Presentation Albumin (g/dL)	Haematuria	Kidney Biopsy	Treatment Received	Clinical Outcome
Alvarado et al. [10] Ecuador	15	M	Not Known	Nil	New-Onset	Anasarca, Dyspnoea, Oliguria	0.55	3.9	1.5	Nil	Not done as inpatient. To be scheduled as outpatient	Chloroquine and Azithromycin, daily boluses of methylprednisolone for 5 doses	Resolution of oedema
Shah et al. [11] United States	8	M	Not Known	Nil	New-Onset	Facial swelling, pedal/scrotal oedema	0.32	11.4	2	Yes, 2+ blood on urinalysis	No	Oral Prednisolone and supportive treatment	Achieved remission, continued oral prednisolone on reporting
Morreale et al. [12] Italy	3	Not Known	Italian, born to non-consanguineous parents	Nil	New-Onset	Abdominal distension/lower limb oedema	Not Known	0.4	1.6	Nil	No	Oral Prednisolone, Intravenous Albumin on Day 1, Furosemide from Day 3	Prednisolone and furosemide were gradually tapered with disease remission
Morgan et al. [13] United States	5	F	Not Known	Nil	New-Onset	Abdominal distension/ lower limb oedema	0.27	>12	2	Nil	No	Intravenous albumin and furosemide for diuresis, oral vitamin D and oral corticosteroids	Achieved complete remission within 3 weeks of starting corticosteroids and urine protein was still negative after 6 weeks of therapy
Basalely et al. [14] United States	Not Known	M	Hispanic	Steroid-sensitive Nephrotic Syndrome with infrequent relapses	Relapse	Anasarca	0.5	18.7	<2.0	Moderate blood, 4–10 RBC, +hyaline casts	No	Received IV Abx. Blood Cultures +ve for Strep. Agalactiae, Stress-dose IV Hydrocortisone followed by oral Prednisolone, IV Albumin and IV Furosemide, prophylactic VTE treatment	Completed 10 days Abx treatment and 2 weeks of prophylactic VTE treatment alongside oral Prednisolone
Enya et al. [15] Japan	3	M	Japanese	Nephrotic Syndrome, Family Hx of Familial Hyper- cholesterolemia	Relapse	Eyelid oedema	0.18	6.3	3.5	Nil	No	Commenced on oral Prednisolone, otherwise supportive management	Achieved remission after a week of treatment
Al-Yazidi et al. [16] Oman	10	M	Arabic (Oman)	Steroid-sensitive Nephrotic Syndrome	Relapse	Facial edema, abdominal distension	Not Known	Not Known	Not Known	Nil	No	Commenced on oral Prednisolone, and required albumin infusion	Tapering of oral Prednisolone dose with resolution of proteinuria
Melgosa et al. [17] Spain (2 patients)		2 patients with steroid-dependent nephrotic syndrome with acute COVID-19 infection provoked a relapse of their nephrotic syndrome. Both patients recovered following administration of oral Prednisolone without complications. Data were not described for each of these 2 patients individually.											
Krishnasamy et al. [18] India (11 patients)		11 out of 24 patients with previous diagnosis of nephrotic syndrome developed relapse of their nephrotic syndrome following acute COVID-19 infection. Data and outcomes were not described for each of these 11 patients individually.											

Abx, antibiotics; COVID-19, coronavirus disease 2019; F, female; IV, intravenous; M, male; RBC, red blood cells; VTE, venous thromboembolism.

**Table 2:** Demographics and outcomes of children and adolescents with glomerulonephritis and other intrinsic kidney pathologies following acute COVID-19 infection.

Author and Country of Report	Age (yrs)	Sex	Ethnicity	Comorbidities	Pathology	New-onset or Relapse	Presentation Creatinine (mg/dL)	Presentation Proteinuria (g/Day)	Presentation Albumin (g/dL)	RBC per High Powered Field	Kidney Biopsy	Treatment Received	Clinical Outcome
Basiratnia et al. [19] Iran	17	M	Not Known	Nil	Acute Necrotising Glomerulonephritis	New-Onset	17	5.6	4	3+ blood in urinalysis, “many” RBCs	Yes	3 doses of pulsed Methylprednisolone followed by Prednisolone and HD with VTE treatment	Discharged DD.
Basiratnia et al. [19] Iran	16	M	Not Known	Nil significant, but episode of gastroenteritis and fever 1 month prior to admission	Acute Necrotising Glomerulonephritis	New-Onset	15.6	Not described. Urinalysis 3+ protein	4	2+ blood in urinalysis, “many” RBCs	Yes	3 doses of pulsed Methylprednisolone followed by Prednisolone and 2 sessions of HD with VTE treatment	Resolution of AKI. Discharged DI.
Fireizen et al. [20] United States	17	M	Not Known	Obesity, Asthma, had COVID-19 pneumonia 2 months prior to presentation	pANCA (MPO) vasculitis	New-Onset	0.78 1st admission, 1.30 2nd admission 1.52 3rd admission, all admissions shortly after one another	Not described	Not stated, but presence of visible proteinuria noted	Not stated, but presence of visible haematuria noted	Yes	Initial admission-Remdesivir, Dexamethasone, and Azithromycin Following vasculitis diagnosis (3rd admission): Methylprednisolone, Plasmapheresis, cyclophosphamide infusions and HD	Resolution Of AKI. Discharged DI.
Meshram et al. [21] India	10	M	Not Known	Nil	Recurrent antifactor H antibody-associated aHUS with underlying complement factor H-related protein mutation	Relapse	2.9	Not described. Urinalysis 3+ protein	Not described	Nil	No	Commenced on oral Prednisolone and MMF. Eventually received IV Rituximab (2 doses) and required IV immunoglobulin as well. Regular antihypertensive medications indicated.	Discharged DD. Patient progressed to CKD eventually and requires maintenance HD. Remained on regular oral Prednisolone

Abx, antibiotics; aHUS, atypical Hemolytic Uremic Syndrome; AKI, acute kidney injury; COVID-19, coronavirus disease 2019; CKD, chronic kidney disease; DD, dialysis dependent; DI, dialysis independent; F, female; HD, hemodialysis; HUS, Hemolytic Uremic Syndrome; IV, intravenous; M, male; MMF, mycophenolate mofetil; MPO, myeloperoxidase; pANCA, perinuclear anti-neutrophil cytoplasmic antibody; VTE, venous thromboembolism.

**Table 3:** Demographics and outcomes of children and adolescents with transplant intrinsic kidney pathologies following acute COVID-19 infection.

Author and Country of Report	Age (yrs)	Sex	Ethnicity	Comorbidities	Pathology	New-Onset or Relapse	Presentation Creatinine (mg/dL)	Presentation Proteinuria (g/Day)	Presentation Albumin (g/dL)	RBC per High Powered Field	Kidney Biopsy	Treatment Received	Clinical Outcome
Levenson et al. [22] United States	16	M	Black	Remote cerebrovascular accident, ESKD secondary to microscopic polyangitis (pANCA vasculitis), live-donor transplant recipient-previous acute antibody rejection	Collapsing Glomerulopathy	New-Onset	2.3 and increasing to 4.7 (baseline 1.5)	17	1.2	Nil	Yes	Acute discontinuation of MMF, required two doses of IV immunoglobulin supportive treatment otherwise	Recovery of graft function, discharged DI, MMF increased back to regular doses
Daniel et al. [23] United States	15	F	Hispanic	ESKD secondary to decreased nephron mass. Patient received deceased donor kidney transplantation	T-cell-mediated rejection	New-Onset	2.1 (baseline is 0.5)	0.31	4	272	Yes	Steroids and Bamlanvimab was administered as post COVID-19 therapy	Discharged with some recovery of graft function.
Berteloot et al. [24] France (2 patients)	2 patients with positive COVID-19 RT-PCR results following kidney transplantation on day 2 and day 105, respectively, were described. Patient 1 had ESKD secondary to HUS, and received a deceased donor transplant. Patient 2 had CKDu, and also received a deceased donor transplant. Transplant kidney biopsy revealed <10% tubular interstitial infiltration in patient 1 and microcalcifications in patient 2. Both patients remained asymptomatic with the positive COVID-19 RT-PCR result.												

COVID-19, coronavirus disease 2019; CKDu, chronic kidney disease of unknown aetiology; DI, dialysis independent; ESKD, end-stage kidney disease; HUS, Hemolytic Uremic Syndrome; MMF, mycophenolate mofetil; pANCA, perinuclear anti-neutrophil cytoplasmic antibody.
